# Assesment of Adulterated Traditional Chinese Medicines in China: 2003-2017

**DOI:** 10.3389/fphar.2019.01446

**Published:** 2019-11-29

**Authors:** Mingzhe Xu, Baobin Huang, Fang Gao, Chenchen Zhai, Yueying Yang, Lulu Li, Wenya Wang, Luwen Shi

**Affiliations:** ^1^Department of Pharmacy Administration and Clinical Pharmacy, School of Pharmaceutical Sciences, Peking University, Beijing, China; ^2^Departmet of General Management, National Institutes for Food and Drug Control, Beijing, China; ^3^School of Pharmaceutical Science, Tsinghua University, Beijing, China; ^4^International Research Center for Medicinal Administration, Peking University, Beijing, China

**Keywords:** traditional Chinese medicines, adulteration, adulterants, supplementary testing methods, analytical methods, drug regulation

## Abstract

Traditional Chinese medicines (TCMs) represent one form of complementary and alternative medicine. The popularity and complexity in production make them attractive and vulnerable to adulteration in stages ranging from planting to production. Adulteration refers to the addition of extraneous, improper, or inferior ingredients that should not be present in TCMs. To detect and combat adulterated TCMs, supplementary testing methods (STMs), which expand the capability of routine testing standards, have been applied in China since 2003. From 2003 to 2017, a total of 184 STMs for TCMs were approved by the Chinese national drug regulatory authority. By assessing these STMs, this research intends to identify those TCMs vulnerable to adulteration, to list common adulterants, and to characterize the techniques of analysis. The results show that adulteration of TCMs can be classified into three main categories: the addition of undeclared drugs/chemical substances, substitution with non-drug components, and the addition of foreign non-drug materials. The top five therapeutic areas of TCMs vulnerable to adulteration are diabetes, calm and sleep, sexual dysfunction, pain relief, and rheumatism. A total of 166 adulterants were detected in the adulterated TCM preparations and herbal products studied here, with 158 adulterants in TCM preparations and 43 in herbal products, with 35 adulterants in common. Each STM consists of different pharmaceutical analysis techniques, including tests for physical-chemical properties, chromatography, spectroscopic techniques, and mass spectrometry. The analytical methodology of STMs relies on the combination of these techniques, with HPLC ranking the highest percentage (76.1%) and physical-chemical techniques the lowest percentage (11.4%). This research shows that STMs have played a crucial role in combating adulterated TCMs. However, STMs represent merely a product testing-centered regulatory strategy. The inspection of cultivation and manufacturing processes should also be strengthened. More importantly, the awareness and self-discipline of TCM manufacturers in implementing good manufacturing practices and regulating the planting and cultivation of raw materials should be improved.

## Introduction

The occurrence and circulation of substandard and falsified medicines is a global problem. World Health Organization (WHO) published two reports in 2017 on the investigation of substandard and falsified medicines and their impact (World Health Organization, 2017). There is no exception of traditional Chinese medicines (TCMs) which are used globally. China exported 455,134 tons of TCMs with a value of almost $4.6 billion USD in 2017 (China Chamber of Commerce for Import and Export of Medicines and Health Products, 2018). In China, TCMs are integrated into China’s healthcare system. TCMs comprise a critical component of the newly revised Chinese essential medicines list (National Health Commission, 2018). In China, TCM laws focus significant attention on the whole lifecycle management of TCMs, ranging from cultivation to manufacturing and distribution (National People’s Congress, 2017). The quality of TCMs including raw herbal products and preparations faces challenges from external factors (Zhang et al., 2012), and the adulteration of TCMs with extraneous chemical substances or banned drugs is a global problem (Ernst, 2002a; Ching et al., 2011; Ching et al., 2018; Ernst, 2002b; Huang et al., 1997). There are three types of adulterations: the addition of undeclared drugs/chemical substances, substitution with non-drug components, and the addition of foreign non-drug materials. With the increase of this phenomenon, the detection of adulterated medicines is becoming increasingly challenging. The intentional adulteration by manufacturers, wholesalers, distributors, and retailers is a form of counterfeiting (National People’s Congress, 2001). It occurs in the whole supply chain ranging from production to distribution. Recently the Chinese drug regulatory authority has paid greater attention to combat counterfeit medicines by cracking down on illegal practices, strengthening site inspection and post-market surveillance (China Food and Drug Administration, 2015), and since 2003, supplementary testing methods (STMs) have been used to detect adulterated TCM (Huang and Xu, 2017). The routine testing methods such as pharmacopeia standards have their limitations and cannot capture those foreign substances which should not be present in medicines (Kopp and Rägo, 2007). The STMs can expand the capability of routine testing methods to detect adulterated TCMs. The study is different from the adulteration program initiated by the American Botanical Council, which is an industry-funded program and addressing adulteration through education rather than federal regulation (The American Botanical Council, 2019).

By investigating the STMs approved from 2003 to 2017, this research intends to provide systematic information regarding TCMs vulnerable to adulterants, list adulterants, and characterize techniques of analysis. Since previously published studies provide limited information regarding the adulteration of TCMs, and global counterfeiting information is inadequate, this research will also contribute to the database of global counterfeit medicines.

## Materials and Methods

The materials of the study are the STMs approved in 2003–2017 (**Supplementary Materials**). The STMs targeting adulterated herbal products and TCMs preparations in forms of pills, tablets, powder, liquid, etc. The STMs are developed and cross-validated by approximately 400 official medicines quality control laboratories nationwide, and reviewed and approved by Chinese drug regulatory authority. A review committee was set up to review the submitted methods with the Secretariat in the China national drug quality control laboratory. The review process is performed using an information management system set up in webpage of national drug quality control laboratory (China National Institutes for Food and Drug Control, 2017a). The system has a database containing all approved STMs. Each approved STM consisted of two parts: the summary note and the method. The note indicates the method developing laboratory, the method validating and verification laboratories, and the application conditions. The method includes adulterants, reference standards, and the combination of different detection techniques, such as simple physico-chemical techniques, thin layer chromatography (TLC), high performance liquid chromatography (HPLC), liquid chromatography mass spectroscopy (LC-MS), and gas chromatography mass spectroscopy (GC-MS). The data of approved STMs is held centrally and can be accessed and retrieved by the official laboratories, regulatory bodies, industries, and consumers nationwide. Statistical analyses were performed on different aspects of the database such as types of adulteration, types of TCM vulnerable to illegal additions, the materials which were illegally added, and the technologies that were used. Each piece of data was double-checked to ensure accuracy. One staff in the Secretariat retrieves data from and transfers to a sheet in the Excel format, another staff checks and confirms each piece of data against each STM. All STMs approved in 2003–2017 were reviewed retrospectively.

## Results

We reviewed all approved STMs on the adulteration of herbal products and TCM preparations. The adulteration can be classified into three categories. A total of 166 adulterants were identified. Concerning a kind of specific TCMs, the number and varieties of adulterants are increasing. Different detection technologies used in STMs were analyzed.

### Types of Adulteration

In general, the adulteration of an herbal product and TCM preparations is for three purposes: the addition of undeclared drugs to render clinical effectiveness (115 methods), the addition of industrial dyes (23 methods), and the substitution to increase weight or quantity (46 methods). The second adulteration scheme includes modifications in approved formulation and/or manufacturing process. This type of adulteration is challenging to detect but is drawing increasing regulatory attention. For example, such adulteration was investigated *via* a site inspection in 2015, and changes in approved manufacturing processes for gingko leaf extracts using hydrochloric acid instead of ethanol to produce hydrolyzed flavones. The hydrolyzed flavone is detected by pharmacopeia standards.

#### Adulteration With Undeclared Drugs to Render Clinical Effectiveness

Drugs, including banned drugs or those with the potential for severe adverse reactions, are often intentionally added to mimic the therapeutic effect of TCMs. Such additions are not stated in the package insert. The therapeutic areas of TCMs vulnerable for adulteration include diabetes, sexual dysfunction, rheumatism, calm and sleep, weight reduction, hypertension, cough and asthma, and pain relief, among others. See **Table 1**.

**Table 1:** The distribution of STMs among therapeutic targets of TCM preparations in 2003-2017.

	Diabetes	Sexual Dysfunction	Rheumatism	Calm/Sleep	Weight reduction	Hypertension	Cough & Asthma	Pain relief	Others*
2003	4	0	2	1	0	0	0	0	2
2004	5	3	0	1	0	1	0	0	0
2005	12	3	3	0	0	0	0	2	1
2006	3	2	2	2	1	1	2	2	0
2007	1	0	0	0	0	0	0	0	4
2008	1	1	2	1	0	0	0	0	0
2009	1	4	1	2	0	2	0	0	0
2010	0	0	0	0	0	0	0	0	0
2011	1	0	0	0	0	1	0	0	0
2012	0	0	0	1	1	0	0	0	0
2013	2	0	0	2	0	0	0	0	0
2014	0	0	1	3	0	1	0	5	3
2015	0	0	0	0	0	0	0	0	1
2016	0	0	0	1	0	0	0	0	3
2017	0	0	0	0	0	0	0	3	11
In total	30	13	11	14	2	6	2	12	25

*indicate skin disease, heart disease, brain, stomach, liver, maternal formulations, among others.

#### Adulteration With Industrial Dyes, and Substitution to Increase Weight or Quantity

Because a particular herbal used for the formulation of TCM preparations has a specific color and appearance, industrial dyes are often used to mimic natural herbals to increase quality by improving the physical appearance. For example, *Carthamus tinctorius* L. was dyed with Orange II and *Coptis chinensis* Franch. was dyed with Auramine O. In some case, the adulteration by dyes cannot be captured by certain official standards. For example, the *Hypericum perforatum* adulterated with dyes can pass USP test (McCutcheon, 2016). As per TCM theory and philosophy, herbal plants consist of both medicinal and non-medicinal parts. To increase the quantity of herbal products, non-medicinal plant parts are added. For instance, the substitution of stem part of *Panax notoginseng* (Burk.) F.H. Chen with its leaf part. And to add sands and other materials in herbals to increase weight.

#### Total Number and Classification of Adulterants

**Figure 1:**
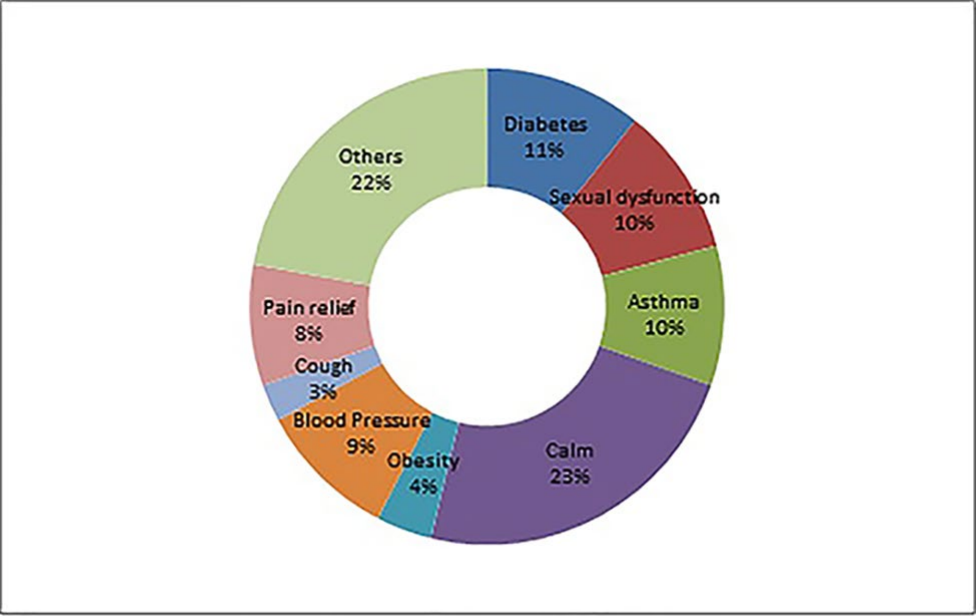
Distribution of adulterants among therapeutic areas.

**Figure 2:**
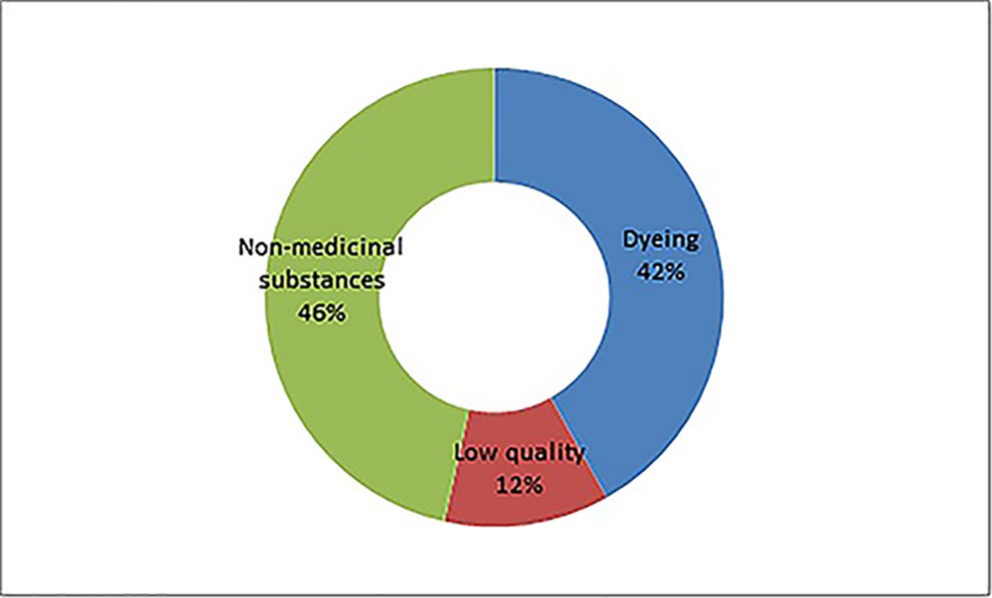
Three categories of adulteration.

A total of 166 adulterants were detected in the adulterated TCM preparations and herbal products, with 158 adulterants found in TCM preparations, and 43 adulterants found in herbal products. There were 35 adulterants in common for both. Concerning frequency, 104 adulterants were detected once, 62 adulterants were detected twice or more. The frequency of adulterants detected by STMs are listed in **Table 2**, with the incidents of detection totaling above four. Concerning the purpose of adulteration, the distribution of adulterants is presented in **Figures 1** and **2**.

**Table 2:** The top seven adulterants identified in herbal and TCM preparations.

ranking	Adulterants	Frequency	Purpose
1	Abietic acid	20	With unofficial part to increase weight
2	Glibenclamide	16	To mimic the function of treating diabetes
	Auramine O	16	Dye* used in herbals to increase quantity
3	Rumex madaio	8	substitution to increase quantity
	808 scarlet	8	dye used in herbals to increase quantity
4	Phenformin	7	To potentiate anti-diabetes effects
5	Diazepam	6	To potentiate sedative effects
	Sildenafil	6	To potentiate effects on sexual dysfunction
	Prednisone	6	To potentiate antitussive and anti-asthmatic effects
	Acetate		
	Total Ash	6	To increase weight
	Orange II	6	Dye used to increase quantity
	Free quercetin	6	To reflect the change of manufacturing process
	Kaempferide	6	To mimic the change of manufacturing process
6	Carmine	5	To dye herbals to increase quantity
	Foreign organic substances	5	To increase weight
7	Paracetamol	4	To potentiate anti-asthmatic effects
	Sibutramine	4	To potentiate weight-loss effects
	Sunset yellow	4	Dye used to increase quantity

*Dyes are used to mask adulterant, nonactive plant material to increase quantity.

### Adulteration Trends

**Figure 3:**
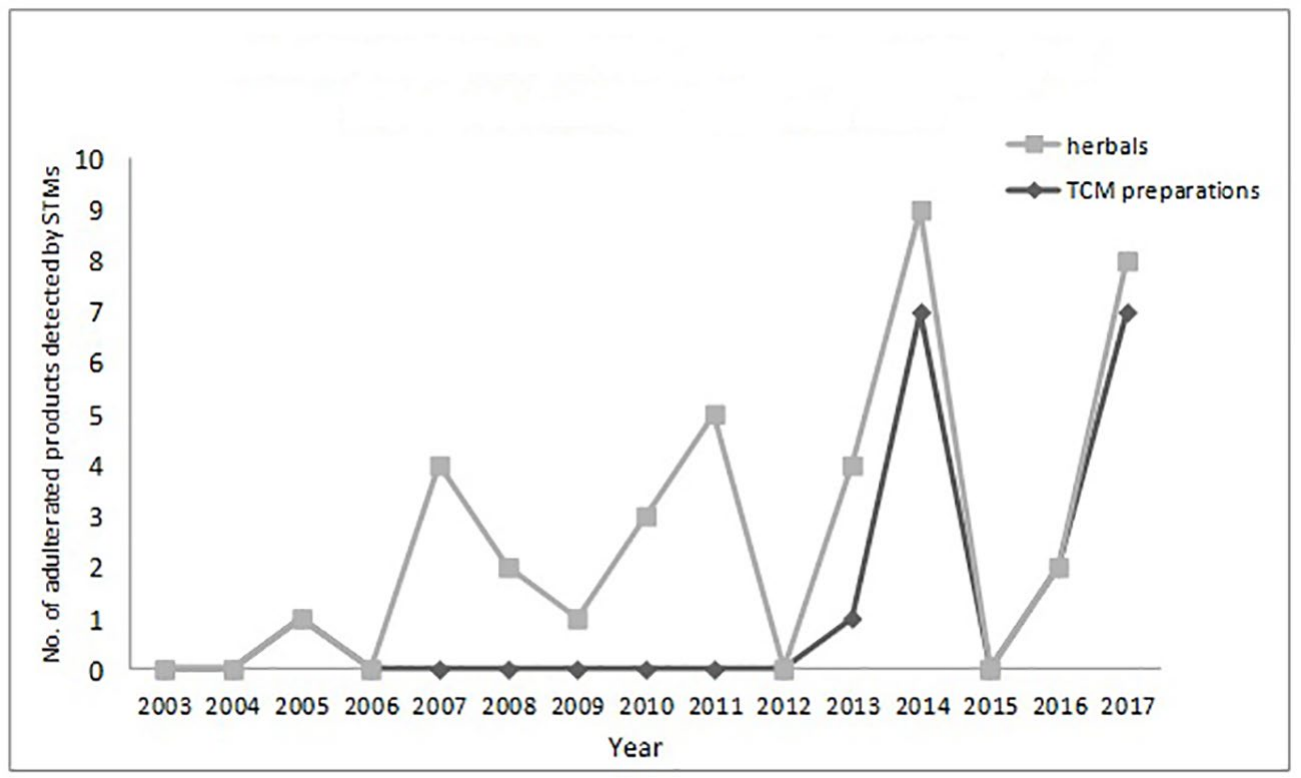
Herbals products and TCM preparations with dyed herbals in between 2003 and 2017.

The numbers and varieties of adulterants in a given TCM are increasing. There is a certain percentage of adulterants that a single STM may detect more than one adulterant. For TCM preparations, there are 145 methods, with 100 methods detecting one adulterant, and with 45 methods (31%) detecting more than one adulterant. For herbals, there are 39 methods, with 23 methods detecting one adulterant, and with 16 methods (41%) detecting more than one adulterants. Regarding therapeutic areas vulnerable to adulterated TCM treatments, the TCM preparations that tend to be most commonly targeted those used for reducing blood glucose levels and enhancing sexual capability. The number and varieties of adulterants detected in these two types of TCM preparations are increasing. Regarding TCM preparations for lowering blood glucose, there was only one adulterant, glibenclamide, detected in 2003; however, in 2009 this number had increased to 11 types of adulterants (10 additional). Regarding TCM preparations for enhancing sexual capability, there were two adulterants, diazepam, and tadalafil, detected in 2004; by 2009 the number of adulterants increased to 12 types (11 additional); see **Table 3**. Regarding the adulterants in herbal preparations, the use of dyes is increasing. For *Carthamus tinctorius L.*, there was one adulterant, orange II, detected in 2007, while three additional dyes were detected in 2013—acid red 73, tartrazine, and carminum. For *Daemonorops draco* Bl., there were two adulterants detected in 2008: tony red IV, scarlet 808, while tony red I was also detected in 2013; see **Table 4**. Furthermore, since 2007, there was an increasing tendency for the detection of dyed herbals, which peaked between 2014 and 2017. This change for adulterated herbals is consistent with trends seen with TCM preparations; see **Figure 3**.

**Table 3:** Chemical adulterants in traditional Chinese medicine preparations used for lowering blood glucose and enhancing sexual functions detected with supplementary testing methods during 2003-2017.

Year	Adulterants in traditional Chinese medicine preparations for lowering blood glucose	Adulterants in traditional Chinese medicine preparations for enhancing sexual functions
2003	glibenclamide	NA
2004	glibenclamide, glipizide, gliclazide	diazepam, tadalafil
2005	glibenclamide, gliclazide, glimepiride, phenformin hydrochloride, metformin hydrochloride	sildenafil citrate, tadalafil
2006	glibenclamide, glipizide, phenformin	sildenafil citrate, sildenafil, tadalafil
2007	glimepiride, gliclazide, gliquidone, pioglitazone, repaglinide, metformin hydrochloride	NA
2008	gliclazide	tadalafil, sildenafil l, acetildenafil, vardenafil, homosildenafil, hydroxyhomo sildenafil, amino tadalafil, pseudo vardenafil
2009	glibenclamide, gliclazide, glipizide, gliquidone, glimepiride, phenformin hydrochloride, metformin hydrochloride, rosiglitazone maleate, repaglinide, pioglitazone hydrochloride, tolbutamide	Methyltestosterone, Acetildenafil, Norneosildenafil, Thioaildenafil, and other 8 kinds of chemical substances which are the same as those in 2008
2010	NA	NA
2011	butylene hydrochloride	NA
2012	NA	NA
2013	glibornuride	NA
2014	NA	NA
2015	NA	NA
2016	NA	NA
2017	NA	NA

NA indicates that adulterants were not listed due to the unavailability of approved methods during this year.

**Table 4:** The herbal medicines and corresponding adulterants detected during 2003-2017.

Year	Adulterated herbal medicines	Adulterants
2003	NA	
2004	NA	
2005	NA	
2006	NA	
2007	typhae pollen, *Scutellariae Radix*	auramine O
	*Cartheami flos*	orange II
	*Schisandrae chinensis fructus*	carmine, erythrosine, acid red
2008	*Cinnabaris*	Scarlet 808
	*draconis sanguis*	rosin, scarlet 808, tony red IV
	AquilariaeLignum Resinatum	rosin
2009	MumeFructus	amaranth red, brilliant blue, sunset yellow
2010	*Coptidis* rhizoma, *Phellodendri chinensis* cortex, *Corydalis rhizoma*	auramine O
2011	Indigo naturalis	malachite green
	*Cordyceps*	amaranth, carmine, sunset yellow, brilliant blue, scarlet 808
	*Olibanum, Myrrha*	rosin
	*dendrobiicaulis, Cuscutae semen*	auramine O
	*Crocistigma*	auramine O, new red, tartrazine, carminum
2012	NA	
2013	*Curcumae longae* rhizoma	orange II, auramine O
	*draconis sanguis*	tony red I, tony red IV, scarlet 808, rosin
	Cartheami Flos	acid red 73, orange II, tartrazine, carminum
2014	*Schisandrae chinensis fructus*	amaranth red, brilliant blue, sunset yellow
	*Cartheami flos*	azorubine, sunset yellow
2015	NA	
2016	*Liquidambaris resina*	rosin
2017	*Cuscutae semen*	tartrazine

### The Technologies Used for Adulterant Detection

Each approved method consisted of a set of technologies to detect adulterants directly or the abnormal levels of existing ingredients. The strategy for developing methods are as follows: each method is a combination of different detection techniques used to measure adulterants quantitatively or qualitatively or measure the abnormal level of ingredients reflected by normal testing standards. The techniques include physico-chemical techniques, TLC, HPLC, LC-MS, and GC-MS. Concerning the sequence of techniques, physico-chemical and TLC detection are used as screening methods and the methods with high sensitivity and specificity used for confirmation. In the 184 STMs, HPLC was most commonly used, in a total of 141 STMs (76.1%) over 15 years. Physico-chemical techniques were used the least frequently, in 21 STMs (11.4%) over 7 years; see **Table 5**.

**Table 5:** Frequency of techniques used to detect adulterants in herbals and TCM preparations in 2003-2017.

Year	No. of STM	Physical-chemical	TLC	HPLC	LC-MS
2003	7	2	6	6	0
2004	10	0	4	9	0
2005	21	0	5	19	13
2006	15	0	5	8	9
2007	13	4	7	5	5
2008	14	4	6	9	8
2009	19	2	10	14	12
2010	9	3	6	5	4
2011	16	0	11	14	9
2012	5	0	4	4	5
2013	8	0	8	8	8
2014	16	0	4	16	14
2015	9	0	0	7	0
2016	6	1	3	5	3
2017	16	5	3	12	12
(total)	184	21	82	141	102

## Discussion and Conclusion

To the authors’ knowledge, our study is the most comprehensive regarding the adulteration of TCMs in China concerning the therapeutic areas and adulterants used. Previous studies showed sexual enhancers, and weight reduction formulations are the most frequently targeted medicines. A strategy of combined detection technologies ranging from screening to confirmation which are similar to our study is adopted. Our study also covers rheumatism, calm and sleep, pain relief, skin diseases, heart disease, brain, stomach, liver, and maternal disease, among others. Compared with a previous systematic study (Almuzain et al., 2013), which reports adulteration with undeclared and synthetic drugs, our study also covers adulteration with chemical dyes. In addition, our study also analyses the characteristics of testing techniques.

This research contributes to the global database concerning the adulteration of TCMs by adding information from China. Before the foundation of the China Food and Drug Administration (CFDA) in 2013, all approved methods were limited to regulatory bodies and its affiliated testing labs at three levels: national, provincial, and city levels. A major concern is the publicity of STM will help those who intend to violate regulation to escape the detection of adulterants. Since the foundation of the CFDA, a philosophy of transparency has been adopted, and all approved STMs should be published for two considerations: consistent testing is threatening to offenders, and will help those TCM preparations manufactures to control outsourced herbals. Furthermore, such publicity provides the possibility for the conducting this research.

For TCM, adulteration is among the extrinsic factors affecting the safety of TCM (Koh and Woo, 2000). Extrinsic adverse effects are largely due to a failure of good manufacturing practices arising from adulteration, substitution, contamination, among other factors. The distribution of adulterated TCMs in rural areas occurred more frequently than that in urban areas due to lack of adequate enforcement resources. External quality issues can be classified into three main categories: cultivation, manufacturing, and circulation (herbal materials and herbal products). In manufacturing stage, the offenders intend to have economic gains by changing manufacturing process, which will bring foreign substances to final preparations. For instance, some illegal manufacturers changed the manufacturing process of *Ginkgo biloba L.* extract with dilute hydrochloric acid instead of dilute ethanol (Liu et al., 2017). It has been reported that the factors of constrained TCM resources, fast-growing demand, a long production chain, underdeveloped production techniques and regulation, and poor awareness of personnel contribute to the occurrence of adulterated TCMs. As raw materials of TCMs preparations grow and are harvested as agricultural products, TCMs might contain adulterants such as heavy metals because of soil contamination (Ernst, 2002). While this type of adulteration is not intentional, it should be considered along with intentional adulteration. For unintentional adulteration, the amounts of adulterants are usually at very low levels and can only be detected by advanced instruments with high sensitivity. For intentional adulteration, the amounts of adulterants are typically found at high levels. Therefore, the detection of adulterants should take a tolerant attitude because the accidental unregulated operation of TCM harvest does exist at present. The implementation of Good Agricultural and Collecting Practice (GACP) could minimize the chance of unintentional adulteration to a large extent.

A limitation of compendia exists regarding the detection of adulterants. Currently, our efforts in upgrading compendia usually focus on ensuring medicines contain what should be present and limiting what unavoidably cannot be removed. The upgraded compendia do not readily account for the risk of accidental or intentional adulteration. As another aspect, the official drug quality standards rely on the conditions of medicine production. Once the conditions are not compliant with Good Manufacturing Practice (GMP) standards, the official standards cannot take effect. Therefore, STM is needed to intentionally detect those adulterants by expanding the capability of official standards.

Detection technologies provide varying degrees of qualitative and quantitative data about medicines. The main categories of techniques for pharmaceutical analysis can be delineated as follows: visual inspection, tests for physical properties, chemical tests, chromatography, spectroscopic techniques, and mass spectrometry. STM relies on the combination of different techniques. Each technique has advantages and disadvantages. For instance, the TLC is cost-saving and easy for operation; however, it has poor sensitivity, specificity, and accuracy as compared with HPLC and MS. The latter is expensive and time-consuming. Thus, the best solution is a complementary method. Typically, the best approach is to work through tests beginning with the easiest or least expensive method and then proceed to the more expensive or difficult tests. It is appropriate to combine techniques to obtain the maximum amount of relevant information for a suspected sample. This combination strategy is in line with the analytical approach used broadly in falsified medicines (Rebiere et al., 2017).

STM, by its nature, is the endpoint of testing and is a testing-centered regulation philosophy. In developing STM, there are several challenges. One is the availability of reference standards. To justify the outcome of testing, reference standards are important as they provide the criteria for component qualities. As per Drug Administrative Law, China Food and Drug Control (NIFDC) is responsible for developing, calibrating, preparing, and distributing reference standards used in compendia, such as pharmacopeia standards. Many compounds which may be detected are not listed in the Chinese pharmacopeia; therefore, reference materials are not always readily available. To solve problems related to the availability of reference materials the NIFDC is preparing reference materials, and is currently launching a digital reference materials initiative (Wang et al., 2016). Secondly, the fast-changing list of common adulterants challenges STM development. Offenders will change adulterants, such as through modification of parent molecules to escape detection *via* STM. Furthermore, the type of adulteration is evolving from the addition of illegal foreign substances to alteration of production processes, such as some TCM herbals are added without further extraction preparation. Third, to solve the problem from its nexus, the development of STM for TCM should not only involve preparation but also control herbal production (raw materials).

To tackle the problem of adulteration in TCM, China has continuously strengthened the supervision of traditional Chinese herbal products and constantly increased the frequency and intensity of production site inspections and post-market surveillance in distribution channels. The inspection and testing results are published, which will effectively reduce illegal behaviors. In 2015, for the illegal practices in the production and distribution of *Ginkgo biloba* L. exact and its preparations, 47 firms were penalized and fined nearly 14.7M USD. Seven responsible persons were forbidden from engaging in work related to the production and distribution of medicines (China Food and Drug Administration, 2015). The adulteration of TCMs is being controlled. The nationwide post-market investigation in China shows that the average rate of compliance with official quality standards for TCMs increases from 64% in 2013 to 77% in 2016 (China Pharmaceutical News, 2017). In 2018, the China national drug regulatory authority released a one-year plan to further strengthen the regulation on TCMs, covering some key trading markets of herbal market such as Ango and Bozhou (China National Medicines and Health Product Administration, 2018). In general, there is growing trend among healthcare professionals to rely on TCMs for the prevention and treatment of chronic diseases (Fan et al., 2018). And the Chinese government released strategic plan for the development of TCMs in healthcare services, which further enables the development of TCM industry (State Council of China, 2016).

However, quality control problems related to traditional Chinese medicines are still not to be underestimated. Many factors may affect the quality of traditional Chinese medicines, such as defects in the herbal materials, deficiencies in the manufacturing process, inadequacies in medicine labeling, among other issues (Li et al., 2018). In addition, the uniform standard system of traditional Chinese medicines is lacking. The standard system of traditional Chinese medicines includes the Chinese pharmacopoeia and regional standards. In the case of prepared slices and crude medicines not listed in the pharmacopeia or other national standard documents, regional standards can be issued by regional governments as a supplement to national standards. As a result, one prepared slices might be qualified in its native region while not in another; this situation is presently stirring debate over the necessity of repealing all regional standards for prepared slices and the need for issuance of a unified national standard (Hu and Calduch, 2017).

Further strengthening of TCM regulations to minimize the risk of adulteration requires rigorous implementation of GACP and GMP (Zhang J. et al., 2012). This includes identification of possible problems in the chain, such as the use of pesticides, fungal toxin formation, diseased plants, the time and season of harvesting, incorrect plant species, postharvest processing, and the drying of raw materials (Chao et al., 2017). Furthermore, enterprises should strengthen their awareness and self-discipline and focus on product quality control. At the same time, some producers can organize themselves into groups and associations to facilitate communication, recommend standard analytical methods, and mprove the quality of products. Finally, traditional Chinese medicines are an inseparable part of a chain in the Chinese medical security scheme and a significant portion of essential Chinese drugs. Addressing and preventing adulterations require enforcement of regulatory systems, increased sampling and monitoring, training of food producers and handlers, and the development of effective, rapid, and cost-effective methods of fraud detection (Tibola et al., 2018). The Chinese government will continue to improve pharmacopeia standards and overcome shortcomings in the field of traditional Chinese medicine. To tackle the circulation of concerned TCMs in global market, the joint efforts given by consumers, industries, and regulatory bodies are required, such as case reporting, audit of TCM firms, and information sharing among regulatory authorities.

There are some limitations inherent to this study. This research aims to evaluate the current situation regarding TCM adulteration. However, we indirectly collected information about approved STMs to detect adulteration. An assumption is that the number of approved STMs represents the severity of TCM adulteration each year to a large extent. The factors which affect the development, review, and approval of STMs can also have an impact on the number of STMs approved each year.

## Author Contributions

Initiated and designed the manuscript: BH, MX. Analyzed data and collected literature: FG, CZ, YY, LL. Wrote the manuscript: BH, MX. WW and LS read and approved the final version.

## Conflict of Interest

The authors declare that the research was conducted in the absence of any commercial or financial relationships that could be construed as a potential conflict of interest.
