# Supplementary material for: Assesment of Adulterated Traditional Chinese Medicines in China: 2003-2017
Source: Front Pharmacol. 2019 Nov 29;10:1446. doi: 10.3389/fphar.2019.01446 (PMC6895211; doi:10.3389/fphar.2019.01446)
Supplement: Supplementary file 1 [file Table_1.docx]

**Table 1: the distribution of STMs among therapeutic targets of TCM preparations in 2003-2017**

|  | Diabetes | Sexual Dysfunction | Rheumatism | Calm/Sleep | Weight reduction | Hypertension | Cough & Asthma | Pain relief | Others*: |
| --- | --- | --- | --- | --- | --- | --- | --- | --- | --- |
| 2003 | 4 | 0 | 2 | 1 | 0 | 0 | 0 | 0 | 2 |
| 2004 | 5 | 3 | 0 | 1 | 0 | 1 | 0 | 0 | 0 |
| 2005 | 12 | 3 | 3 | 0 | 0 | 0 | 0 | 2 | 1 |
| 2006 | 3 | 2 | 2 | 2 | 1 | 1 | 2 | 2 | 0 |
| 2007 | 1 | 0 | 0 | 0 | 0 | 0 | 0 | 0 | 4 |
| 2008 | 1 | 1 | 2 | 1 | 0 | 0 | 0 | 0 | 0 |
| 2009 | 1 | 4 | 1 | 2 | 0 | 2 | 0 | 0 | 0 |
| 2010 | 0 | 0 | 0 | 0 | 0 | 0 | 0 | 0 | 0 |
| 2011 | 1 | 0 | 0 | 0 | 0 | 1 | 0 | 0 | 0 |
| 2012 | 0 | 0 | 0 | 1 | 1 | 0 | 0 | 0 | 0 |
| 2013 | 2 | 0 | 0 | 2 | 0 | 0 | 0 | 0 | 0 |
| 2014 | 0 | 0 | 1 | 3 | 0 | 1 | 0 | 5 | 3 |
| 2015 | 0 | 0 | 0 | 0 | 0 | 0 | 0 | 0 | 1 |
| 2016 | 0 | 0 | 0 | 1 | 0 | 0 | 0 | 0 | 3 |
| 2017 | 0 | 0 | 0 | 0 | 0 | 0 | 0 | 3 | 11 |
| In total | 30 | 13 | 11 | 14 | 2 | 6 | 2 | 12 | 25 |

Note * indicates kin disease, heart disease, brain, stomach, liver, maternal formulations, among others.
