# Supplementary material for: Assesment of Adulterated Traditional Chinese Medicines in China: 2003-2017
Source: Front Pharmacol. 2019 Nov 29;10:1446. doi: 10.3389/fphar.2019.01446 (PMC6895211; doi:10.3389/fphar.2019.01446)
Supplement: Supplementary file 2 [file Table_2.docx]

**Table 2. The top seven adulterants identified in herbal and TCM preparations**

| ranking | Adulterants | Frequency | Purpose |
| --- | --- | --- | --- |
| 1 | Abietic acid | 20 | With unofficial part to increase weight |
| 2 | Glibenclamide | 16 | To mimic the function of treating diabetes |
|  | Auramine O | 16 | Dye* used in herbals to increase quantity |
| 3 | Rumex madaio | 8 | substitution to increase quantity |
|  | 808 scarlet | 8 | dye used in herbals to increase quantity |
| 4 | Phenformin | 7 | To potentiate anti-diabetes effects |
| 5 | Diazepam | 6 | To potentiate sedative effects |
|  | Sildenafil | 6 | To potentiate effects on sexual dysfunction |
|  | Prednisone Acetate | 6 | To potentiate antitussive and anti-asthmatic effects |
|  | Total Ash | 6 | To increase weight |
|  | Orange II | 6 | Dye used to increase quantity |
|  | Free quercetin | 6 | To reflect the change of manufacturing process |
|  | Kaempferide | 6 | To mimic the change of manufacturing process |
| 6 | Carmine | 5 | To dye herbals to increase quantity |
|  | Foreign organic substances | 5 | To increase weight |
| 7 | Paracetamol | 4 | To potentiate anti-asthmatic effects |
|  | Sibutramine | 4 | To potentiate weight-loss effects |
|  | Sunset yellow | 4 | Dye used to increase quantity |

Note *Dyes are used to mask adulterant, non active plant material to increase quantity.
