# Supplementary material for: Assesment of Adulterated Traditional Chinese Medicines in China: 2003-2017
Source: Front Pharmacol. 2019 Nov 29;10:1446. doi: 10.3389/fphar.2019.01446 (PMC6895211; doi:10.3389/fphar.2019.01446)
Supplement: Supplementary file 3 [file Table_3.docx]

Table 3. Chemical adulterants in Traditional Chinese Medicine preparations used for lowering blood glucose and enhancing sexual functions detected with supplementary testing methods during 2003-2017

| year | Adulterants in traditional Chinese medicine preparations for lowering blood glucose | Adulterants in Traditional Chinese Medicine preparations for enhancing sexual functions |
| --- | --- | --- |
| 2003 | glibenclamide | NA |
| 2004 | glibenclamide, glipizide, gliclazide | diazepam, tadalafil |
| 2005 | glibenclamide, gliclazide, glimepiride, phenformin hydrochloride, metformin hydrochloride | sildenafil citrate, tadalafil |
| 2006 | glibenclamide, glipizide, phenformin | sildenafil citrate, sildenafil, tadalafil |
| 2007 | glimepiride, gliclazide, gliquidone, pioglitazone, repaglinide, metformin hydrochloride | NA |
| 2008 | gliclazide | tadalafil, sildenafil l, acetildenafil, vardenafil, homosildenafil, hydroxyhomo sildenafil, amino tadalafil, pseudo vardenafil |
| 2009 | glibenclamide, gliclazide, glipizide, gliquidone, glimepiride, phenformin hydrochloride, metformin hydrochloride, rosiglitazone maleate, repaglinide, pioglitazone hydrochloride, tolbutamide | Methyltestosterone, Acetildenafil, Norneosildenafil, Thioaildenafil, and other 8 kinds of chemical substances which are the same as those in 2008 |
| 2010 | NA | NA |
| 2011 | butylene hydrochloride | NA |
| 2012 | NA | NA |
| 2013 | glibornuride | NA |
| 2014 | NA | NA |
| 2015 | NA | NA |
| 2016 | NA | NA |
| 2017 | NA | NA |

**Note:** NA indicates that adulterants were not listed due to the unavailability of approved methods during this year
