# Supplementary material for: Assesment of Adulterated Traditional Chinese Medicines in China: 2003-2017
Source: Front Pharmacol. 2019 Nov 29;10:1446. doi: 10.3389/fphar.2019.01446 (PMC6895211; doi:10.3389/fphar.2019.01446)
Supplement: Supplementary file 4 [file Table_4.docx]

Table 4 The herbal medicines and corresponding adulterants detected during 2003-2017

| Year | Adulterated herbal medicines | Adulterants |
| --- | --- | --- |
| 2003 | NA |  |
| 2004 | NA |  |
| 2005 | NA |  |
| 2006 | NA |  |
| 2007 | typhae pollen, *Scutellariae* *Radix* | auramine O |
|  | *Cartheami flos* | orange II |
|  | *Schisandrae chinensis fructus* | carmine, erythrosine, acid red |
| 2008 | *Cinnabaris* | Scarlet 808 |
|  | *draconis sanguis* | rosin, scarlet 808, tony red Ⅳ |
|  | AquilariaeLignum Resinatum | rosin |
| 2009 | MumeFructus | amaranth red, brilliant blue, sunset yellow |
| 2010 | *Coptidis* rhizoma, *Phellodendri chinensis* cortex, *Corydalis rhizoma* | auramine O |
| 2011 | Indigo naturalis | malachite green |
|  | *Cordyceps* | amaranth, carmine, sunset yellow, brilliant blue, scarlet 808 |
|  | *Olibanum, Myrrha* | rosin |
|  | *dendrobiicaulis, Cuscutae semen* | auramine O |
|  | *Crocistigma* | auramine O, new red, tartrazine, carminum |
| 2012 | NA |  |
| 2013 | *Curcumae longae* rhizoma | orange Ⅱ, auramine O |
|  | *draconis sanguis* | tony red I, tony red Ⅳ, scarlet 808, rosin |
|  | Cartheami Flos | acid red 73, orange II, tartrazine, carminum |
| 2014 | *Schisandrae chinensis* *fructus* | amaranth red, brilliant blue, sunset yellow |
|  | *Cartheami flos* | azorubine, sunset yellow |
| 2015 | NA |  |
| 2016 | *Liquidambaris resina* | rosin |
| 2017 | *Cuscutae semen* | tartrazine |
