# Supplementary material for: Assesment of Adulterated Traditional Chinese Medicines in China: 2003-2017
Source: Front Pharmacol. 2019 Nov 29;10:1446. doi: 10.3389/fphar.2019.01446 (PMC6895211; doi:10.3389/fphar.2019.01446)
Supplement: Supplementary file 5 [file Table_5.docx]

**Table** 5 Frequency of techniques used to detect adulterants in herbals and TCM preparations in 2003-2017

| **Year** | **No. of STM** | **Physical-chemical** | **TLC** | **HPLC** | **LC-MS** |
| --- | --- | --- | --- | --- | --- |
| **2003** | **7** | **2** | **6** | **6** | **0** |
| **2004** | **10** | **0** | **4** | **9** | **0** |
| **2005** | **21** | **0** | **5** | **19** | **13** |
| **2006** | **15** | **0** | **5** | **8** | **9** |
| **2007** | **13** | **4** | **7** | **5** | **5** |
| **2008** | **14** | **4** | **6** | **9** | **8** |
| **2009** | **19** | **2** | **10** | **14** | **12** |
| **2010** | **9** | **3** | **6** | **5** | **4** |
| **2011** | **16** | **0** | **11** | **14** | **9** |
| **2012** | **5** | **0** | **4** | **4** | **5** |
| **2013** | **8** | **0** | **8** | **8** | **8** |
| **2014** | **16** | **0** | **4** | **16** | **14** |
| **2015** | **9** | **0** | **0** | **7** | **0** |
| **2016** | **6** | **1** | **3** | **5** | **3** |
| **2017** | **16** | **5** | **3** | **12** | **12** |
| **(total)** | **184** | **21** | **82** | **141** | **102** |
